# Multistage pathogenesis and disease associations of *Mycobacterium avium* subsp. *paratuberculosis*

**DOI:** 10.3389/fimmu.2026.1948490

**Published:** 2026-09-15

**Authors:** Ruoxi Tao, Wanying Zheng, Ling Peng, Shuojie Lv, Qingjing Wang

**Affiliations:** 1 Key Laboratory of Artificial Organs and Computational Medicine of Zhejiang Province, Key Laboratory of Pollution Exposure and Health Intervention of Zhejiang Province, Shulan International Medical College, Zhejiang Shuren University, Hangzhou, Zhejiang, China; 2 Hangzhou Cining Hospital, Hangzhou, Zhejiang, China

**Keywords:** autoimmune diseases, Crohn’s disease, *Mycobacterium avium* subsp. *paratuberculosis* (MAP), pathogenesis, strain typing, virulence factors

## Abstract

*Mycobacterium avium* subsp. *paratuberculosis* (MAP) is the primary causative agent of Johne’s disease (JD) in ruminants and bears potential public-health implications. Cumulative epidemiological and molecular evidence implies MAP may contribute to Crohn’s disease (CD), whereas its links to multiple sclerosis (MS), type 1 diabetes mellitus (T1DM), and amyotrophic lateral sclerosis (ALS) remain associative without proven causality. This narrative review synthesizes MAP environmental distribution, strain genotyping, transmission routes, and multistage pathogenic mechanisms. MAP breaches the intestinal barrier via fibronectin attachment protein (FAP)-mediated invasion of microfold cells and establishes intracellular persistence within macrophages. It deploys virulence factors including protein kinase G (PknG) to inhibit phagosome–lysosome fusion, the stringent response regulator RelA for metabolic reprogramming, and the ESX-3 secretion system for iron acquisition, while subverting host immunity through M2 macrophage polarization and interleukin-10 (IL-10)/interleukin-6 (IL-6)-mediated anti-inflammatory responses, ultimately driving chronic granulomatous inflammation and tissue injury. We also critically assess diagnostic limitations—particularly the inability of polymerase chain reaction (PCR) to distinguish viable from non-viable organisms—and highlight major research challenges, including extrapolation of virulence mechanisms from other mycobacterial species. This work provides a theoretical framework for prevention, diagnosis, and targeted intervention of MAP-associated disorders.

## 1 Introduction

*Mycobacterium avium* subsp. *paratuberculosis* (MAP) is an acid-fast, weakly Gram-positive bacillus. It is primary causative agent of Johne’s disease (JD), also known as paratuberculosis (PTB), in ruminants. MAP has attracted considerable scientific attention worldwide, Owing to its major impact on livestock production and its growing relevance to public health. MAP belongs to the *Mycobacterium avium* complex (MAC), which comprises four subspecies: *M. avium* subsp. *hominissuis* (MAH), *M. avium* subsp. *avium* (MAA), *M. avium* subsp. *silvaticum* (MAS), and MAP (1, 2). Based on current molecular typing methods, MAP strains are broadly classified into three major genotypes: sheep type (S type; type I/III), cattle type (C type; type II), and bison type (B type) (3, 4). Among these, S type strains, represented by isolates such as Telford and JIII-386, exhibit marked host specificity and are predominantly isolated from sheep and goats, with generally showing lower virulence. In contrast, C type strains, including the reference strain K-10 and the vaccine strain 316F, have a broad host range and infect a wide variety of domestic and wild animal. Although B type strains, represented by the Korean isolate MAPK_JJ1, were initially identified in bison, they have also shown a high prevalence among cattle populations from specific geographic regions. Despite high genetic homology between B-type and C-type strains, the B type forms a distinct phylogenetic sub-branch nested within the broader C-type lineage (5–7)(**Figure 1**).

**Figure 1:**
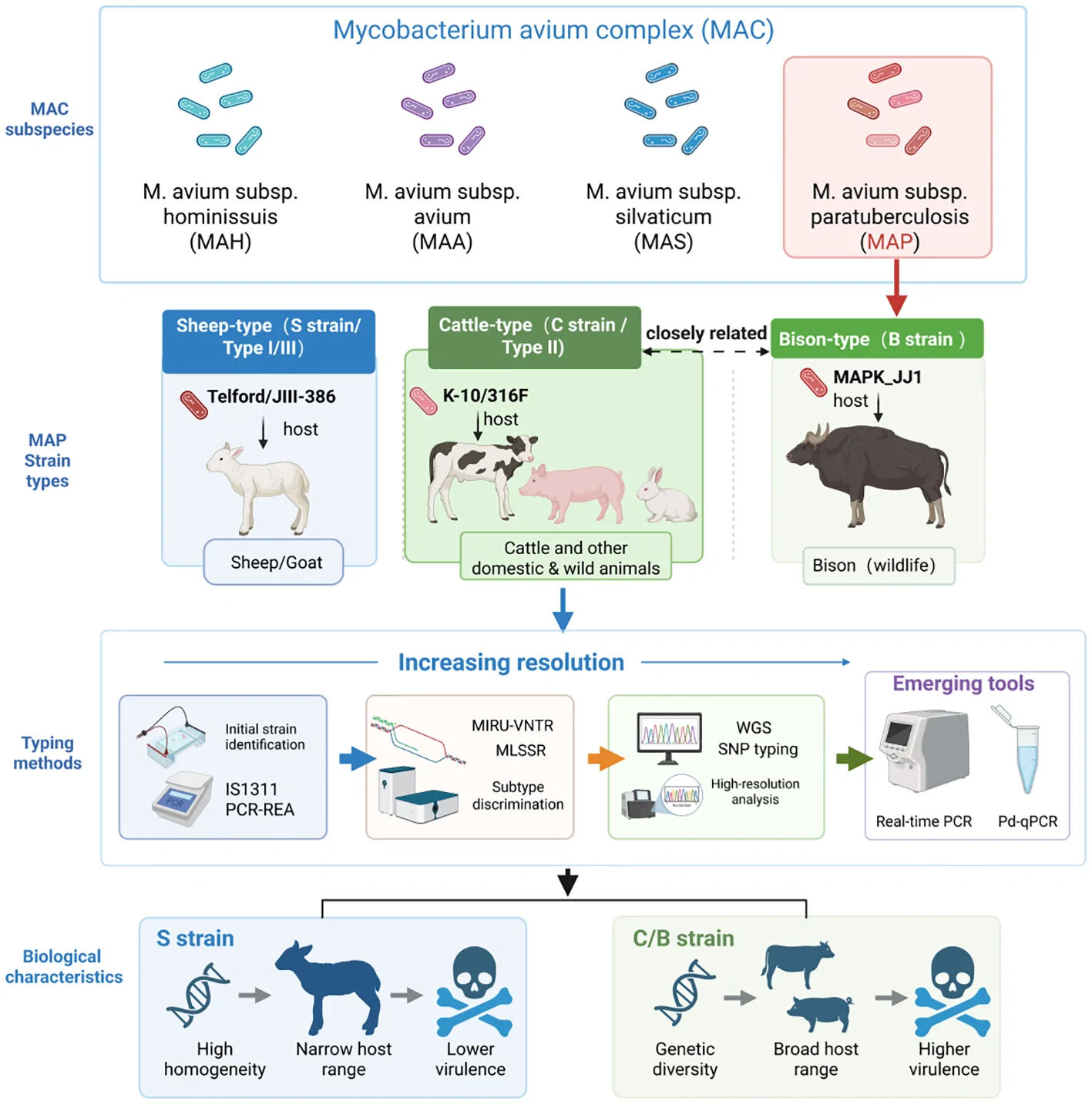
Classification, typing methods, and biological characteristics of *Mycobacterium avium* subsp. paratuberculosis (MAP). The *Mycobacterium avium* complex (MAC) comprises four subspecies, with MAP as the causative agent of Johne’s disease (JD) in ruminants. MAP is classified into three major strain types: sheep type (S type/Type I–III), cattle type (C type/Type II), and bison type (B type). Representative strains include Telford and JIII-386 for the S type, K-10 and 316F for the C type, and MAPK_JJ1 for the B type. The S type presents greater genetic homogeneity, a narrower host range, and lower virulence, whereas the C/B type presents greater genetic diversity, broader host adaptability, and greater virulence. Molecular typing methods for MAP include insertion sequence-based assays (IS1311 PCR-REA), tandem repeat analyses (MIRU-VNTR and MLSSR), whole-genome sequencing (WGS), SNP typing, and emerging tools such as real-time PCR and Pd-qPCR, with increasing discriminatory resolution.

MAP infection is now globally widespread and is recognized as one of the most economically significant chronic infectious diseases in the livestock industry. A systematic review covering 48 countries reported that herd-level prevalence exceeded 20% in approximately half of the surveyed countries, whereas prevalence rates surpassed 40% in several developed nations (8). National serological surveillance in the United States revealed an overall MAP positivity rate of 21.6% in dairy herds, which reached 39.7% in large-scale farms with ≥300 cattle, suggesting a significant positive correlation between herd size and infection risk (7). Beyond cattle, MAP infects a broad range of domestic ruminants, notably sheep, deer, and camels. MAP infection in wildlife species, particularly deer, bison, wild boars, and other free-ranging animals, has been reported in approximately 38% of countries worldwide, thus establishing natural reservoirs that increase the risk of interspecies transmission (9).

Epidemiologically, livestock are most susceptible to MAP infection before two years of age, particularly during the first few weeks after birth. Transmission occurs predominantly via the fecal–oral route, although vertical transmission via contaminated milk and transplacental infection have also been documented. Clinically infected animals typically show chronic diarrhea, progressive weight loss, and reduced productivity. Although disease progression is generally slow, about 10–20% of infected animals eventually succumb to advanced-stage disease, causing substantial economic losses in the agricultural sector (8). Furthermore, MAP demonstrates remarkable environmental persistence and can survive for extended periods under adverse conditions, thereby contaminating dairy products and posing threats to both animal husbandry and public health (9). A comprehensive understanding of MAP biology and pathogenesis is therefore essential for improving disease surveillance, prevention strategies, and therapeutic development.

In recent years, accumulating evidence has indicated that MAP not only is an animal pathogen but also may contribute to the pathogenesis of several human autoimmune and neurodegenerative disorders, notably Crohn’s disease (CD), multiple sclerosis (MS), type 1 diabetes mellitus (T1DM), and amyotrophic lateral sclerosis (ALS). These links have raised growing concerns about the broader public health implications of MAP infection. This review comprehensively summarizes recent advances in the understanding of the environmental distribution, transmission routes, molecular typing characteristics, pathogenic mechanisms, and disease associations. We also discuss major challenges and future research directions, providing a theoretical framework for disease prevention, mechanistic studies, and the development of targeted therapeutic strategies.

## 2 *Mycobacterium avium* subsp. *paratuberculosis*

### 2.1 Environmental distribution and routes of infection

MAP exhibits remarkable tolerance to environmental stressors, including acidic conditions, elevated temperatures, and high salinity (10). These adaptive characteristics enable MAP to persist for prolonged periods in diverse environmental reservoirs, including soil, feces (10), and aquatic environments, particularly surface water and drinking water supplies (11–13). MAP is transmitted primarily through contaminated feed, milk, and water. However, the bacterial load in feces from infected cattle is substantially greater than that detected in milk, suggesting that fecal–oral transmission is the predominant route of infection. Following fecal–oral transmission, MAP crosses the intestinal epithelial barrier by preferentially targeting microfold (M) cells (14). This process is mediated by the bacterial fibronectin attachment protein (FAP), which binds host fibronectin (FN) and subsequently interacts with β1 integrins, expressed on the apical membrane of microfold cells(M cells), facilitating bacterial internalization and transepithelial translocation (14). Once internalized by macrophages, MAP establishes long-term intracellular persistence by inhibiting phagosome–lysosome fusion, a key immune evasion mechanism that promotes survival within host cells (10, 15).

Beyond direct host-to-host transmission (16), MAP shed in the excreta of infected animals can remain environmentally stable for extended periods in soil, dust, and fecally contaminated water systems (10). Like other potential zoonotic pathogens, MAP originating from livestock environments may enter rivers and surface water systems through rainfall runoff (17), contaminating domestic water supplies (11, 18) and posing difficult to quantify public health risks to human populations (13)(**Figure 2**).

**Figure 2:**
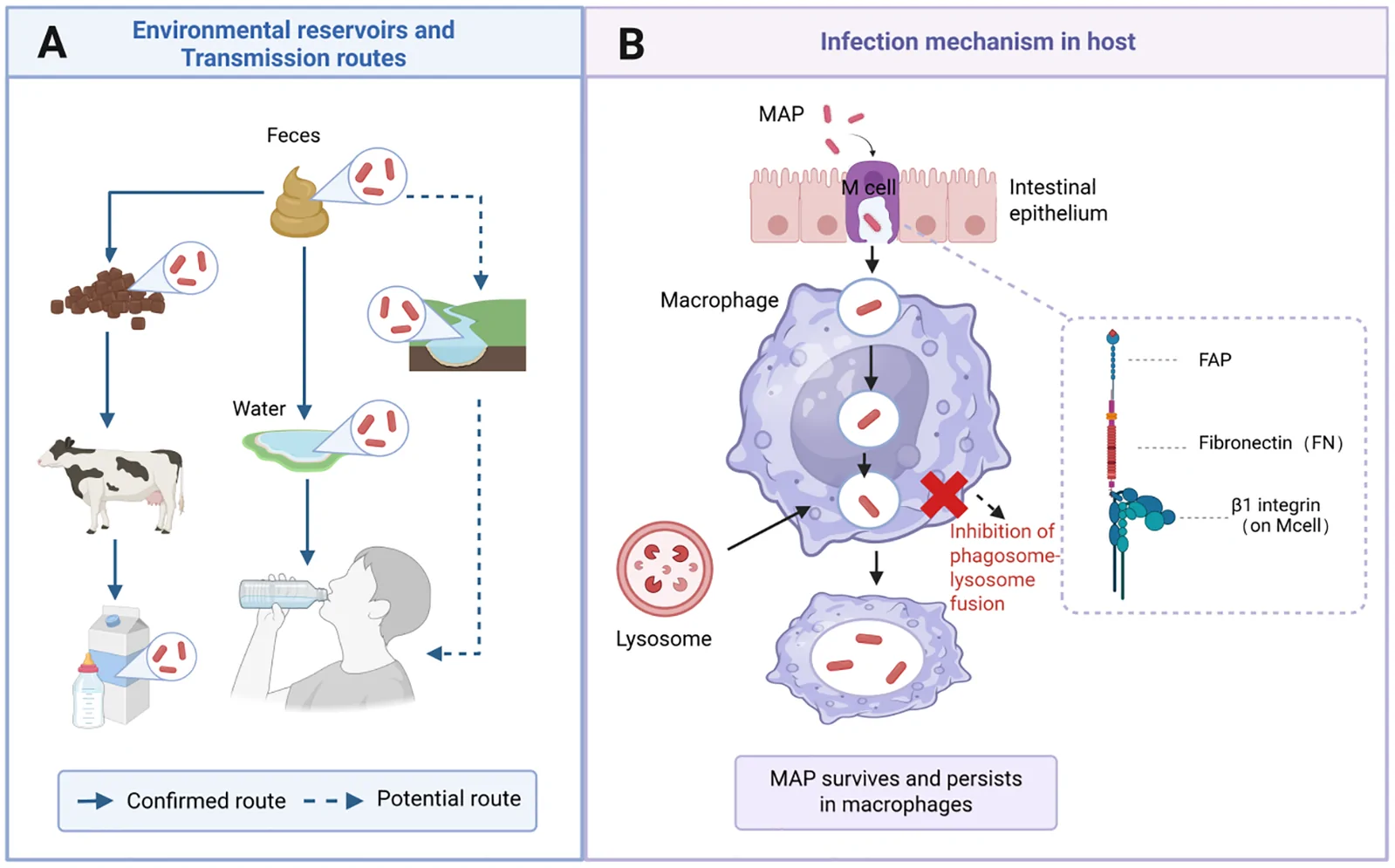
Environmental reservoirs, transmission routes, and initial infection mechanisms of MAP. **(A)** MAP persists in animal feces, soil, water, and dairy products. Transmission occurs via confirmed fecal-oral routes to animals and potential contamination of food and water to humans. **(B)** MAP preferentially invades the intestinal epithelium through microfold cells (M cells). Its fibronectin attachment protein (FAP) interacts with host fibronectin (FN) and β1 integrin on M-cell surfaces to facilitate internalization. Following macrophage uptake, MAP inhibits phagosome–lysosome fusion, enabling long-term intracellular survival.

### 2.2 Strain typing and identification techniques

MAP strains are broadly classified into three principal genotypes: C type, S type, and B type (19). Current strain-typing methodologies include insertion sequence-based approaches, chiefly Insertion sequence 1311 (IS1311) polymerase chain reaction (PCR)-restriction endonuclease analysis (PCR-REA); tandem repeat-based methods, notably mycobacterial interspersed repetitive unit–variable number tandem repeat (MIRU-VNTR) analysis and multilocus short sequence repeat (MLSSR) typing (20); and whole-genome analytical strategies, including pangenome analysis and single-nucleotide polymorphism (SNP)-based genotyping (21). Collectively, these techniques have evolved from conventional lineage discrimination methods to high-resolution subtyping platforms capable of identifying fine-scale genomic variation among isolates (22). Among currently available methods, MLSSR and whole-genome sequencing (WGS)-based analyses provide the highest discriminatory power, enabling detailed characterization of strain-specific genetic diversity (20) and potential virulence-associated traits (21). More recently, emerging approaches such as real-time PCR-based genotyping and palladium compound treatment combined with quantitative polymerase chain reaction (qPCR), the Pd-qPCR viability assay, have further improved the specificity, sensitivity, and practical applicability of MAP detection and strain typing (23). These advances provide increasingly powerful tools for molecular epidemiological surveillance, transmission tracing, and the development of targeted disease control strategies (24)(**Figure 1**).

## 3 MAP-associated diseases

### 3.1 Johne’s disease

Johne’s Disease (JD) is a chronic granulomatous enteritis of ruminants caused by MAP. MAP infection is considered one of the most economically significant bacterial diseases affecting the dairy industry worldwide (10). Following oral exposure, MAP colonizes the gastrointestinal tract of young calves (25), with infection primarily localized to the distal ileum (26), where the pathogen establishes long-term persistence (27).

Clinically, infected cattle often maintain a normal appetite despite progressive disease development. However, infection ultimately leads to reduced milk production, chronic diarrhea, progressive emaciation, and substantial production losses (10). The progression of JD is generally divided into three distinct stages. The first stage is a characterized by a prolonged latent phase following infection in juvenile animals, during which MAP remains undetectable via conventional diagnostic methods. The second stage is characterized by subclinical infection, during which MAP can be detected via fecal antigen testing or serological assays such as enzyme-linked immunosorbent assay (ELISA) (28). During the third stage, animals exhibit overt clinical signs accompanied by detectable humoral immune responses and extensive bacterial shedding (29–31).

Importantly, infected livestock continuously shed MAP through feces and milk (10), facilitating long-term environmental dissemination and transmission to susceptible animals (32). Thus, management strategies aimed at reducing early-life exposure are critical for disease control. For example, rearing calves separately from adult cattle (33) and minimizing exposure to MAP-contaminated dust (34) or manure have been shown to significantly reduce the risk of infection (35)(**Figure 3**).

**Figure 3:**
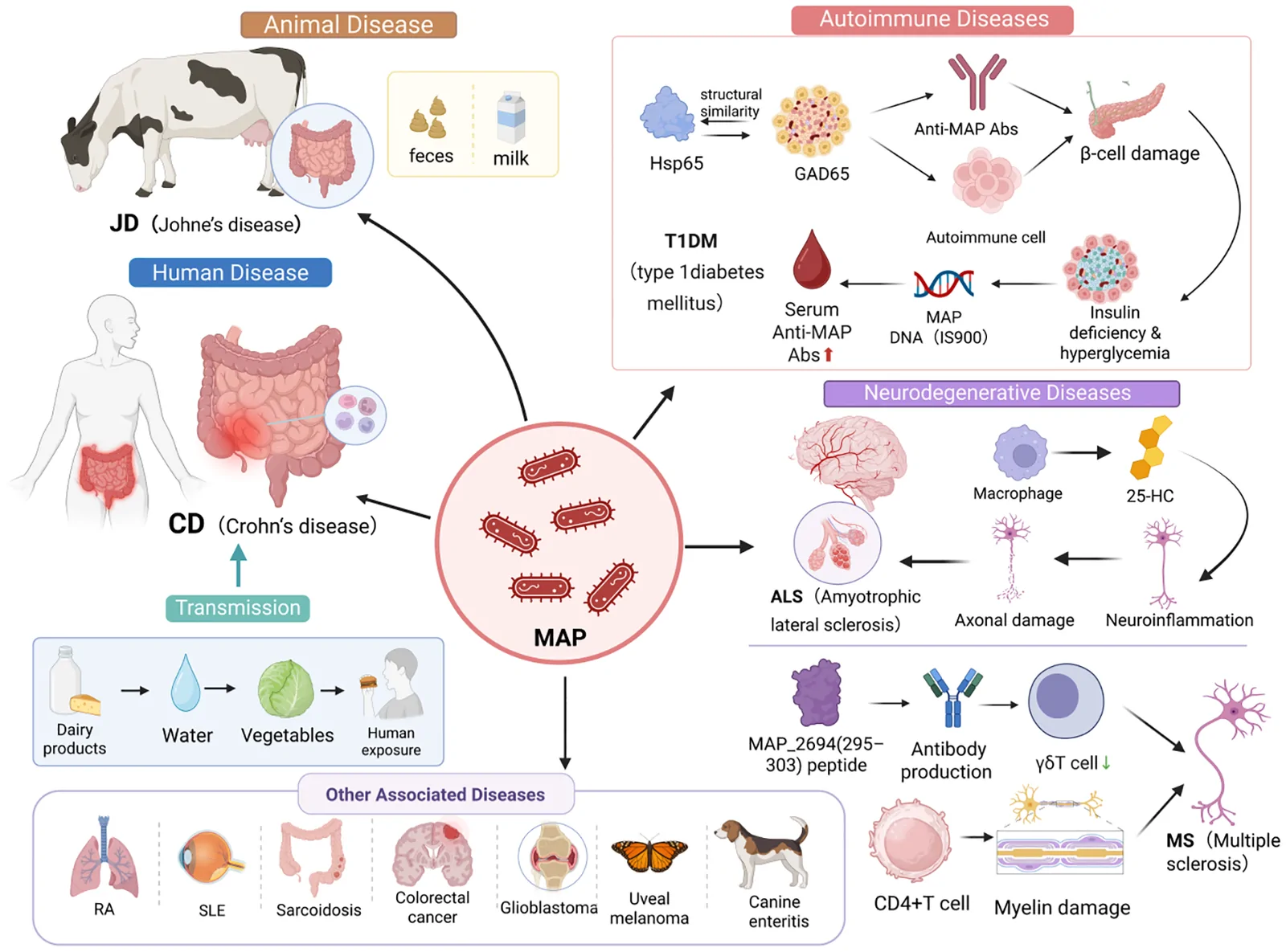
Diseases associated with MAP infection. MAP causes chronic granulomatous enteritis, known as Johne’s disease (JD), in ruminants and is epidemiologically linked to multiple human disorders. It is a potential environmental trigger for Crohn’s disease (CD), with similar pathological granulomas. MAP-derived heat shock protein 65 (Hsp65) shares structural mimicry with human glutamic acid decarboxylase 65 (GAD65), driving β-cell destruction, insulin deficiency, and hyperglycemia in type 1 diabetes mellitus (T1DM); elevated blood glucose is accompanied by increased serum anti-MAP antibodies, which can be detected via MAP-specific diagnostic assays. Macrophages infected with MAP produce neurotoxic 25-hydroxycholesterol (25-HC), which contributes to axonal injury and neurodegeneration in amyotrophic lateral sclerosis (ALS). MAP-specific peptides may deplete γδ T cells, promoting myelin damage in multiple sclerosis (MS). Additional associations include rheumatoid arthritis (RA), systemic lupus erythematosus (SLE), sarcoidosis, colorectal cancer, glioblastoma, uveal melanoma, and canine enteritis.

### 3.2 Crohn’s disease

CD is a chronic inflammatory disorder of the gastrointestinal tract that predominantly affects the terminal ileum. Common clinical manifestations include malaise, weight loss, abdominal pain, and chronic diarrhea. Histologically, CD is characterized by granulomatous inflammation, a pathological feature that closely resembles lesions observed in JD and other mycobacterial diseases (36, 37).

Evidence suggests a potential association between MAP and the pathogenesis of CD. Compared with patients with ulcerative colitis (UC) or individuals with suspected inflammatory bowel disease, MAP has been isolated more frequently from patients with CD (38–40), consistent with regional epidemiological surveys (36). Moreover, MAP RNA has been detected in intestinal biopsy samples from patients with CD (41)36, an observation corroborated by population-based studies (36). In addition, host immune responses directed against MAP antigens (36), together with the reported efficacy of antimycobacterial therapies in subsets of patients with CD, further support a potential contributory role for MAP in disease pathogenesis (42–45).

Nevertheless, the role of MAP in CD remains controversial. Critics of the MAP causation hypothesis argue that MAP may represent an environmental organism or intestinal commensal rather than a primary pathogen and that CD may instead arise from dysregulated immune responses driven by molecular mimicry among MAP antigens, the gut microbiota, and host intestinal tissues (36, 46, 47). Consistent with this skepticism, large-scale randomized controlled trials of antimycobacterial combination therapies, including the RHB-104 trial and earlier regimens combining clarithromycin, rifabutin, and clofazimine, have shown only limited or non-sustained clinical benefits in CD patients, despite encouraging preliminary observational signals (48, 49). Meta-analyses and systematic reviews have also highlighted substantial inter-study heterogeneity: MAP detection rates by PCR or culture vary greatly across CD patient cohorts and different geographic regions (50). Of note, low but non-negligible rates of MAP DNA have also been reported in stool samples from healthy individuals and patients with UC. This background positivity complicates straightforward case-control interpretations and cautions against attributing CD pathogenesis solely to MAP infection (9, 51).

Human exposure to MAP is believed to occur primarily through the consumption of contaminated food or water, including inadequately pasteurized milk, dairy products, fecally contaminated vegetables, contaminated beef, and drinking water (10). Notably, MAP has been detected in both unpasteurized and commercial pasteurized milk as well as milk products (52), and viable organisms have been cultured from bulk tank milk (53).Several studies have demonstrated that conventional milk pasteurization procedures may not completely eliminate MAP, likely because MAP is more heat resistant than the microorganisms currently used to establish pasteurization standards, such as *Mycobacterium bovis* and *Coxiella burnetii* (52). Furthermore, potable water has been implicated as a potential transmission route based on epidemiological investigations of urban CD clusters (54), and highly specific real-time PCR assays have confirmed MAP contamination in human fecal and intestinal samples (55).These findings underscore the importance of rigorous food sterilization and hygiene practices for reducing potential MAP exposure and associated disease risk (**Figure 3**).

### 3.3 Putative links between MAP and neurological disorders

Beyond classical gastrointestinal and systemic inflammatory disorders, MAP exposure has been hypothetically associated with autoimmune and degenerative neurological diseases, including MS and ALS (56). MS is a chronic immune-mediated demyelinating disorder of the CNS characterized by autoreactive lymphocyte infiltration and myelin damage. Serological studies have detected elevated anti-MAP antibody levels in MS patients from geographically confined cohorts, particularly Sardinian and Japanese populations, relative to healthy controls (57, 58). Mechanistically, structural homology between MAP epitopes and human CNS myelin proteins supports a molecular mimicry mechanism, suggesting that persistent MAP exposure may trigger cross-reactive autoimmunity and exacerbate CNS inflammatory injury (56). At the cellular level, the MAP protein MAP2694 shares sequence homology with the T-cell receptor γ-chain (59), and antibodies against its immunodominant epitope MAP_2694295–303 are enriched in MS patients (60), suggesting a potential link to aberrant γδ T-cell responses. Furthermore, MAP may cooperatively promote autoimmunity with Epstein–Barr virus through shared antigenic targets recognized in both serum and cerebrospinal fluid (61). Nevertheless, these associations are population-specific and poorly reproducible in multi-ethnic cohorts. To date, no genomic or histological evidence from human CNS specimens has verified a direct MAP pathogenic role in MS, rendering this association tentative (9).

By contrast, the potential link between MAP and ALS remains largely speculative and relies on limited case-based evidence (62). ALS is a fatal neurodegenerative disease driven by progressive loss of upper and lower motor neurons. Two atypical cases exhibited ALS-like motor weakness accompanied by non-classic inflammatory manifestations, including low-grade fever. Treatment with antimycobacterial antibiotics alleviated both motor and inflammatory symptoms in these individuals, providing tentative clinical clues that mycobacterial infection may contribute to motor neuron injury in specific patient subsets (62). However, definitive MAP infection was not microbiologically confirmed in these cases, and symptomatic improvement may result from nonspecific anti-inflammatory effects of antibiotics rather than targeted MAP elimination. Moreover, no large cohorts, animal experiments or independent validations have corroborated this hypothesis (9). Although direct mechanistic evidence from MAP-exposed neural tissue is absent, it has been speculated that chronic MAP-driven inflammation may contribute to axonal dysfunction, and that MAP-infected macrophages could generate neurotoxic oxysterols such as 25-hydroxycholesterol(25-HC), which is elevated in ALS and has been implicated in motor neuron injury through disruption of neurotrophic signaling (63, 64). Overall, the MAP-ALS association lacks robust causal and reproducible evidence (**Figure 3**).

### 3.4 Diabetes mellitus

The link between MAP and diabetes mellitus appears to be highly subtype specific. Current evidence suggests that MAP may act as an environmental trigger for autoimmune forms of diabetes, especially T1DM and latent autoimmune diabetes in adults (LADA), whereas no significant relationship has been established between MAP infection and type 2 diabetes mellitus (T2DM).

The principal pathogenic mechanism is thought to involve molecular mimicry–mediated autoimmunity. Specifically, several MAP-derived proteins, including heat shock protein 65 (Hsp65), share structural homology with glutamic acid decarboxylase 65 (GAD65), a major autoantigen expressed in pancreatic β cells. Consequently, immune responses directed against MAP antigens may cross-react with host β cells, leading to impaired insulin secretion and development of T1DM. This hypothesis is supported by multiple lines of evidence. MAP-specific DNA sequences, particularly the insertion sequence 900 (IS900), have been detected in peripheral blood samples from T1DM patients. In addition, elevated serum antibody titers against MAP antigens such as MAP3738c and MAP3865c have been reported in affected individuals (60, 65) (**Figure 3**).

By contrast, patients with T2DM show no significant differences in anti-MAP antibody levels or MAP DNA detection rates compared with healthy controls, suggesting that insulin resistance-driven metabolic dysfunction in T2DM patients is unlikely to be linked to MAP infection (66). Case-control studies performed in Sardinian cohorts found comparable seropositivity for MAP antigens among T2DM patients and non-diabetic controls, with low positive rates that were statistically indistinguishable from healthy subjects (67). A subsequent meta-analysis of available observational data further confirmed the absence of a statistically significant association between MAP exposure and T2DM, despite broad confidence intervals that could not fully exclude weak effects in specific populations (68). Taken together, these observations suggest that MAP-driven pathogenic effects are largely restricted to autoimmune-mediated β-cell injury, and do not contribute to the metabolic pathophysiology characteristic of T2DM (66).

### 3.5 Additional MAP-related diseases

Beyond JD in ruminants, MAP exposure has been hypothetically associated with multiple human and animal disorders. However, most of these correlations come from limited studies and lack consistent independent replication; to date, no definitive causal evidence identifies MAP as an independent disease-causing agent (9).

In humans, MAP has been proposed as a potential environmental trigger for sarcoidosis, a multisystem inflammatory disease characterized by the abnormal accumulation of immune cells in affected organs. Chronic MAP-induced inflammation may contribute to disease pathogenesis in susceptible individuals (56). Nevertheless, this hypothesis is primarily supported by a single research group and lacks consistent validation in independent patient cohorts (9).

Similarly, epidemiological observations have linked MAP exposure to an increased risk of uveal melanoma, particularly among individuals with prolonged exposure to agricultural environments, such as farmers. Environmental sources, including contaminated soil, dust, and water, have been proposed as potential routes of exposure, and persistent inflammation induced by MAP infection may contribute to tumorigenesis (56, 69).These associations remain preliminary and require external verification (9).

MAP has also been tentatively implicated in colorectal cancer. Sporadic detection of MAP DNA in human colorectal tumors and MAP-induced goblet cell hyperplasia suggest a potential pro-tumorigenic role of chronic intestinal inflammation. However, MAP detection in colorectal tissues shows substantial inter-laboratory inconsistency, and the reproducibility of this association remains poor (9).

Evidence further suggests a possible association between MAP exposure and glioblastoma. The higher incidence rates observed in rural populations, livestock workers, and certain athletic groups have led to speculation that environmental exposure to MAP may contribute to tumor development through mechanisms involving endothelial proliferation and pseudopalisading necrosis, both of which are characteristic histopathological features of glioblastoma (56, 70).Of note, this association relies solely on epidemiological correlation and has not been validated by independent investigations (9).

In autoimmune diseases such as RA and systemic lupus erythematosus (SLE), MAP is considered a putative infectious cofactor rather than a confirmed pathogen. MAP-derived molecular mimicry and altered human endogenous retrovirus K (HERV-K) activity have been proposed to drive autoimmunity in RA and SLE (56).Nevertheless, existing clinical data are inconsistent, and cross-cohort validation is lacking (9).

In veterinary medicine, MAP may contribute to canine chronic enteropathy. One clinical study detected MAP-specific DNA in intestinal biopsies from 19% of dogs with chronic gastrointestinal symptoms (71). However, this single observation has not been replicated in independent canine cohorts, and the pathogenic role of MAP in canine disease remains unconfirmed (**Figure 3**).

### 3.6 Diagnostic approaches

Accurate diagnosis of MAP infection remains challenging, as no single diagnostic modality can reliably detect all infected cases. Existing approaches differ substantially in sensitivity, specificity, cost, clinical applicability, and operational complexity (36). Current MAP detection strategies fall into two major frameworks: direct molecular detection via PCR and fecal culture combined with molecular confirmation, with additional support from serological assays (36).

Fecal culture is traditionally regarded as the gold standard for MAP diagnosis, as it enables direct isolation of viable organisms. This method involves inoculating fecal samples onto mycobactin J–supplemented selective media, such as 7H10 agar, Herrold’s egg yolk medium (HEY), or Lowenstein–Jensen medium, followed by prolonged incubation for 10–16 weeks and, in some cases, up to seven months. Because colony morphology alone cannot reliably distinguish MAP from other mycobacterial species, definitive identification requires subsequent IS900/ISMav2-targeted PCR or 16S rRNA sequencing (36). Although fecal culture offers very high specificity, its major limitations include prolonged turnaround time, technical complexity, high cost, and relatively low sensitivity, particularly in samples with low bacterial burden or mixed microbial contamination (72).

PCR-based methods have become the most widely used molecular approach for MAP detection. Most assays target IS900, a highly specific multicopy genomic element present in about 14–20 copies in the MAP genome (73, 74). Optimized conventional PCR and real-time quantitative PCR (qPCR) assays enable direct amplification of MAP DNA from fecal, tissue, and environmental samples without prior culture, with reported detection limits as low as 122 fg (74). Critically, PCR detects MAP-specific DNA fragments rather than intact viable MAP pathogens. A positive PCR signal only indicates the presence of target nucleic acid sequences; it cannot differentiate genetic material originating from dead degraded bacterial remnants versus replication-competent live MAP organisms. This intrinsic limitation complicates clinical interpretation, as DNA may persist long after bacterial death (73). Moreover, PCR assays are vulnerable to false-positive and false-negative results caused by inefficient DNA extraction, the presence of inhibitory substances in fecal samples, or cross-reactivity with homologous genetic sequences (73).

Among the available diagnostic tools, ELISA is the most widely used method for large-scale screening of MAP infection in livestock owing to its simplicity, low cost, and suitability for high-throughput applications (72, 75, 76). ELISA can detect MAP-specific antibodies in serum or milk samples and is therefore particularly useful for herd-level surveillance. However, its sensitivity depends strongly on the infection stage. During early infection, low antibody titers frequently lead to false-negative results, whereas cross-reactivity with other mycobacterial species may reduce specificity compared with PCR or fecal culture (72, 75, 76).

Salivary biomarker analysis has emerged as a promising noninvasive diagnostic strategy that may complement conventional serological approaches. Pathogen-oriented salivary testing mainly employs IS900-targeted PCR to amplify MAP-specific DNA from saliva specimens. Pilot studies in MAP-exposed ruminants successfully amplified MAP DNA from saliva samples; nevertheless, diagnostic agreement between salivary PCR and gold-standard fecal assays remains poor (77). Importantly, salivary MAP-DNA positivity cannot confirm viable MAP pathogens. Salivary nucleic-acid signals may originate from gastrointestinal contamination rather than authentic salivary-gland infection, and residual DNA from dead bacteria can yield false-positive amplification results (77).

Host-derived salivary protein signatures have also been explored for early-stage infection screening. The levels of functional proteins such as lactoferrin (LF) and lactoperoxidase (LPO) are significantly reduced in the saliva of MAP-infected cattle, potentially preceding detectable antibody responses. These findings suggest that salivary biomarkers may offer a rapid and practical approach for early field-based detection of MAP infection (78). Spectroscopy-driven salivary metabolic profiling has also achieved high accuracy in internal cross-validation, although diagnostic performance declines markedly under external independent testing (79). Despite these encouraging preliminary observations, salivary-based assays still face notable practical obstacles. No unified standard protocols for saliva collection, preservation and pre-processing have been established for MAP diagnosis. Candidate salivary markers, whether microbial nucleic acids or host-response proteins, lack large-scale independent cohort validation across diverse animal populations. At present, salivary biomarkers remain largely exploratory and cannot replace fecal culture or fecal PCR for definitive MAP diagnosis, though they hold potential as preliminary screening tools under field conditions (9) (**Table 1**).

**Table 1:** Characteristics and applications of commonly used diagnostic methods for MAP infection.

Identification method	Detection principle	Advantages	Limitations	Application	Reference
IS900 PCR	Detection of MAP-specific IS900 sequence	High sensitivity and specificity; Rapid	Lack of standardized protocols; false positives from cross-reactivity; DNA quality–dependent; fecal inhibitors	Confirmatory diagnosis; Fecal/tissue detection	(36, 55, 73, 74)
Fecal Culture	Isolation and identification of viable MAP using selective media.	Traditional gold standard; Very high specificity	Time-consuming; Low sensitivity; Labor-intensive	Definitive diagnosis; Research use	(36, 40, 72)
ELISA	Detection of MAP antibodies	Low cost; High throughput	Low early sensitivity; Cross-reactivity	Herd screening; Field surveillance	(28, 72, 75)
Salivary Biomarkers	Detection of LF/LPO alterations	Non-invasive; Early detection potential	Limited validation; Unstandardized	Emerging field diagnostics	(77–79)

MAP, *Mycobacterium avium* subsp. *Paratuberculosis*; IS900, Insertion Sequence 900; PCR, Polymerase Chain Reaction; ELISA, Enzyme-Linked Immunosorbent Assay; LF, Lactoferrin; LPO, Lactoperoxidase.

## 4 Virulence factors and pathogenesis

MAP pathogenesis represents a coordinated multistage process including mucosal invasion, intracellular colonization, immune evasion, and progressive tissue injury, which collectively drive the pathological manifestations observed in JD in ruminants, while putative links to human disorders such as CD remain mechanistically suggestive without direct *in-vivo* human validation.

### 4.1 Invasion and colonization

MAP infection is initiated by breaching the intestinal mucosal barrier and evading early host immune surveillance. Through highly specific interactions between bacterial adhesins and host cell receptors, MAP is translocated from the intestinal lumen into macrophages in a directional manner.

#### 4.1.1 Targeting mucosal invasion of the intestinal epithelium

MAP preferentially invades subepithelial compartments via microfold (M) cells located over Peyer’s patches in the distal ileum (14). M-cell β1-integrin receptors interact with the bacterial fibronectin-attachment-protein (FAP), forming a fibronectin molecular bridge that facilitates transcytosis across the epithelium; this invasion mechanism is supported by direct experimental evidence obtained in MAP using enteroid infection assays (14).

The MAP3464 (35-kDa protein family) activates host Cdc42 signaling to trigger actin rearrangement and membrane ruffling, promoting epithelial cell internalization of MAP, supported by direct MAP infection experiments (80). Mycobacterial cell entry protein(Mce)1A and Mce1D further enhance epithelial and organoid invasion phenotypes, as demonstrated in MAP *in-vitro* infection models (81–83)FAP, MAP3464 and Mce family factors mediate MAP translocation across intestinal barriers, the initial step for granulomatous enteritis in ruminant JD. For human CD patients, if viable MAP colonizes gut mucosa, these same invasion effectors would enable epithelial penetration and trigger local gut inflammation; however, direct human-tissue experimental proof is absent (84)(**Figure 4**).

**Figure 4:**
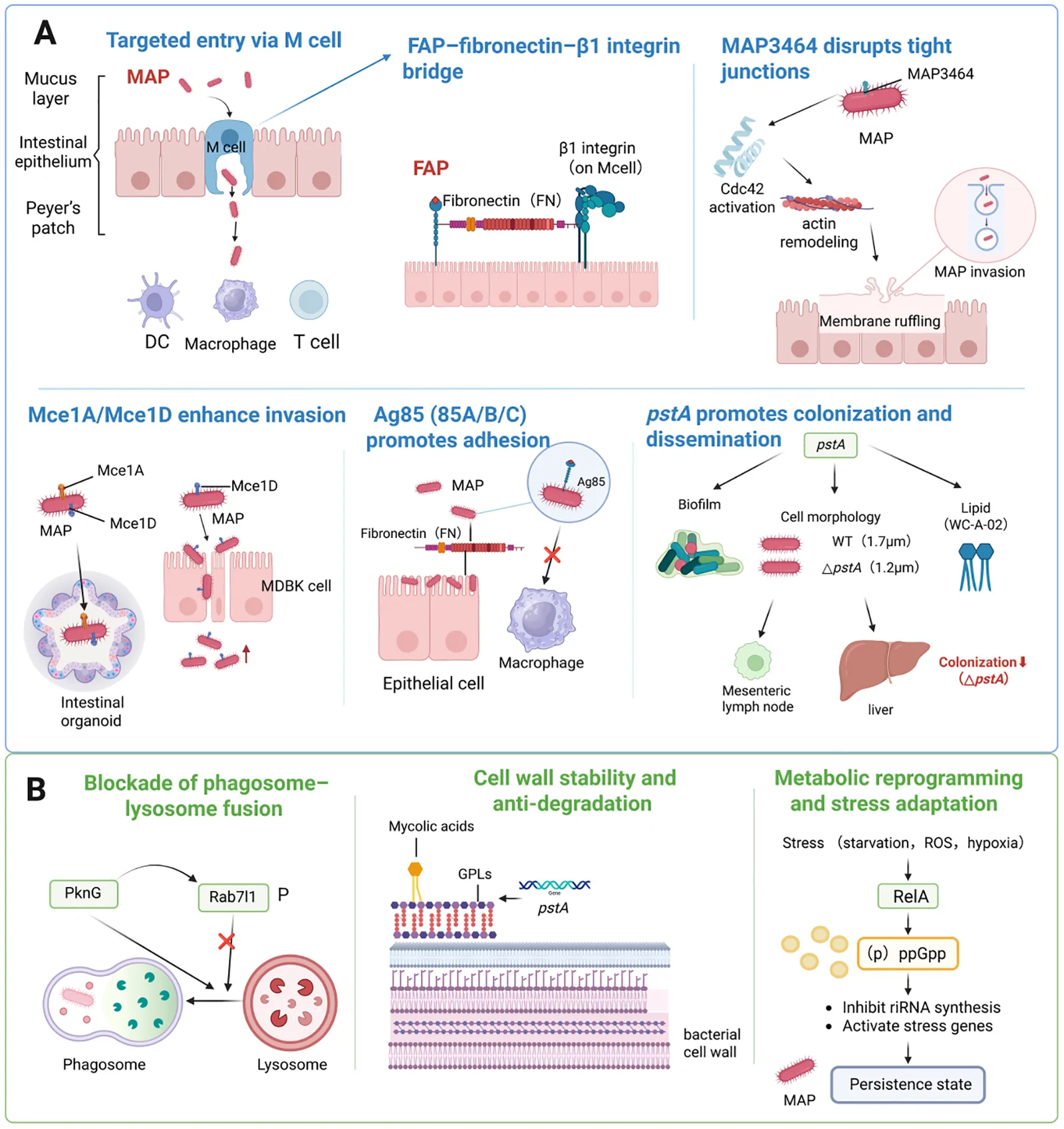
Mechanisms of MAP intestinal invasion, colonization, and intracellular persistence **(A)** Invasion and colonization: MAP preferentially targets microfold cells (M cells) in Peyer’s patches via the FAP–FN–β1 integrin axis, enabling specific adherence and transcytosis across the intestinal epithelium. The MAP3464 protein disrupts tight junctions and activates the host Cdc42 signaling pathway, triggering actin cytoskeleton remodeling and membrane ruffling to facilitate bacterial internalization. Mycobacterial cell entry proteins (Mce1A/Mce1D) enhance epithelial and endothelial invasion, whereas the antigen 85 complex (Ag85) mediates initial adhesion and participates in mycolic acid biosynthesis. The *pstA* gene regulates biofilm formation, cell morphology, and lipid synthesis, promoting intestinal colonization and systemic dissemination to mesenteric lymph nodes and the liver. **(B)** Intracellular persistence: After macrophage uptake, serine/threonine protein kinase G (PknG) phosphorylates host Rab-7-like protein 1(Rab7l1), blocking phagosome–lysosome fusion and preventing bacterial degradation. Mycolic acids and glycopeptidolipids (GPLs) maintain cell wall hydrophobicity and structural integrity, resisting lysosomal enzymes and acidic stress. The *relA* gene encodes RelA, which synthesizes guanosine tetra-/pentaphosphate ((p)ppGpp) under nutrient deprivation or oxidative stress, driving global metabolic reprogramming, suppressing ribosomal synthesis, and activating stress-responsive genes to enter a viable but non-replicative persistent state.

#### 4.1.2 Surface adhesion and colonization enhancement

The antigen 85 complex (Ag85), which includes Ag85A, Ag85B, and Ag85C, is a member of the fibronectin-binding protein family, enhances initial MAP adhesion to intestinal epithelial cells and macrophages through interactions with the host FN. Besides mediating adhesion, Ag85 participates in mycolic acid biosynthesis, and thus indirectly contributes to bacterial resistance to lysosomal degradation. These phenotypes are supported by direct data from MAP recombinant-protein and infection assays (27, 83)(**Figure 4**).

Mycobacterial major-membrane-protein (MMP), previously identified as an immunogenic molecule in *Mycobacterium leprae*(*M.leprae*), is transcriptionally up-regulated in MAP under the hypoxic or osmotic stress conditions characteristic of the intestinal microenvironment, a profile observed in MAP transcriptomic datasets (85). Functional assays using bovine cell models further show that MMP contributes to the invasion of bovine epithelial cells and may also participate in antigen presentation and signaling interactions involving CD4+ and CD8+ T cells, modulating early host immune responses (80, 82, 85). Such activities may facilitate the onset of granulomatous enteritis during ruminant JD; by extension, if MAP colonizes human gut mucosa in CD patients, MMP could hypothetically reshape mucosal antigen presentation and T-cell activation, though direct human-tissue experimental evidence supporting this pathological role remains absent (84).

#### 4.1.3 Genetic regulators of colonization

The *pstA* gene encodes a nonribosomal peptide synthetase that regulates biofilm formation, bacterial morphology, and synthesis of the unique lipopeptide WC-A-02 required for cell-membrane integrity; these phenotypes are supported by direct phenotypic observations from targeted MAP mutant experiments (86, 87). Wild-type MAP strains form structurally organized biofilms, whereas *pstA*-deficient mutants show severely impaired biofilm-forming capacity. Deletion of *pstA* also alters bacterial morphology, shortening cell length from approximately 1.7 μm to 1.2 μm, which may compromise cell-division efficiency and adhesive properties. In bovine experimental infection models, *pstA*-knockout mutants exhibit a greater than 60 % reduction in colonization within mesenteric lymph nodes and hepatic tissue relative to wild-type MAP, pointing to a critical role for *pstA*-dependent traits in systemic dissemination after intestinal barrier penetration (86, 87). Such biofilm-associated adhesive properties and enhanced *in-vivo* organ colonization capacity contribute to the establishment of persistent infection and granulomatous lesions during ruminant JD. By analogy, sustained biofilm-supported MAP persistence would theoretically promote prolonged mucosal exposure in CD if MAP colonizes human intestinal tissue, yet no human-derived experimental data currently substantiate this hypothetical mechanism (**Figure 4**).

### 4.2 Intracellular survival

Following phagocytosis by macrophages, MAP establishes a protected intracellular niche by actively inhibiting phagosome maturation, reinforcing cell wall integrity, and undergoing extensive metabolic reprogramming.

#### 4.2.1 Inhibition of phagosome–lysosome fusion

Serine/threonine protein kinase G (PknG), encoded by the *pknG* gene, is a characterized virulence-associated protein of MAP (27). By analogy to mechanistic findings in *Mycobacterium tuberculosis(*MTB) (52), PknG interferes with normal phagosom-lysosome fusion via phosphorylation-dependent modification of host endosomal trafficking machinery (88); its primary target is Rab-7-like protein 1 (Rab7l1, also designated RAB29, gene *RAB7L1*), a Rab-like GTPase (89), rather than canonical Ras-related protein Rab-7a (Rab7, gene *RAB7A*) *(*90). Although the deletion of *pknG* in MTB results in rapid lysosomal degradation, MAP Δ*pknG* mutants retain partial intracellular survival capability and remain capable of establishing infection in natural hosts (91). This observation implies that MAP may compensate for the loss of PknG function through redundant adaptive pathways, including metabolic regulation mediated by stringent response regulator A (RelA) (92)(**Figure 4**). These PknG-dependent effects support macrophage intracellular survival for granuloma formation in ruminant JD, while a hypothetical role in CD remains without direct human-tissue experimental evidence.

#### 4.2.2 Cell wall structure and resistance to degradation

Mycolic acids, synthesized in part via Ag85-mediated pathways (93), are indispensable lipid constituents of the MAP cell wall (27). These molecules confer pronounced hydrophobicity and structural rigidity, protecting MAP against acidic environments and enzymatic degradation in macrophage lysosomes,findings supported by MAP mutant-derived observations (87). Such cell-wall-driven resistance favors intracellular survival (83) and granulomatous lesion formation during ruminant JD (94).

Glycopeptidolipids (GPLs), which are regulated by the pstA gene that encodes a polyketide synthase-associated protein, also contribute to intracellular survival (27). pstA mutants exhibit reduced survival in macrophages (95), likely due to increased surface hydrophilicity and consequent susceptibility to immune detection and elimination phenotypes obtained from direct MAP mutant assays (87), with GPL biosynthetic machinery initially characterized in M. smegmatis (96)(**Figure 4**). This GPL-mediated survival advantage supports persistent macrophage infection in JD.

#### 4.2.3 Metabolic rewiring and stress adaptation

RelA, encoded by the *relA* gene, synthesizes guanosine tetraphosphate/pentaphosphate [(p)ppGpp], a central mediator of the bacterial stringent response (27). Under nutrient deprivation or oxidative stress conditions characteristic of the phagosomal environment of macrophages, RelA promotes a persistent survival state by suppressing ribosomal biosynthesis and activating stress-responsive genes (97), with MAP relA mutant studies confirming this phenotype (91).

Δ*relA* mutants fail to establish persistent intestinal infection in calves and are eventually eliminated by host immunity (92); this *in-vivo* attenuation phenotype was directly obtained from MAP experimental infection assays (91) supporting the critical contribution of *relA* to immune evasion and the maintenance of chronic infection (52)(**Figure 4**).RelA-dependent stringent response enables long-term MAP persistence driving progressive JD pathology.

Additional metabolism-associated genes, including 2-C-methyl-D-erythritol 2,4-cyclodiphosphate synthase (*gcpE*), potassium-transporting ATPase subunit C (*kdpC*), 3-oxoacyl-[acyl-carrier-protein] reductase (*fabG2_2*), inositol monophosphatase A (*impA*), and nucleoid-associated protein Lsr2 (*lsr2*), were uncovered in a large-scale Tn5367 transposon-insertion mutant screen of MAP. Transposon-disrupted mutants of *gcpE*, *kdpC*, *fabG2_2*, and *impA* show impaired macrophage survival and attenuated tissue colonization in mouse infection models, placing candidate intracellular-persistence factors. Notably, these results originate from insertional mutagenesis without genetic complementation, and experiments were conducted in mice rather than natural ruminant hosts (83, 95).Although *lsr2* has been successfully deleted in MAP using targeted allelic-exchange mutagenesis, no published macrophage or in-vivo infection phenotypes are currently available for *Δlsr2* mutants (91). Together, these loci are thought to contribute to MAP intracellular metabolic homeostasis and stress resistance during infection.

### 4.3 Immune suppression and evasion

MAP achieves long-term persistence through sophisticated modulation of host immune responses, including suppression of macrophage bactericidal activity, induction of immune tolerance, and skewing of adaptive immune polarization.

#### 4.3.1 Subversion of macrophage function

MAP suppresses phagosomal acidification, lysosomal degradation, and phagosome–lysosome fusion through multiple, including dysregulation of host pre-mRNA alternative splicing (98). For example, aberrant splicing of the *monocyte-to-macrophage differentiation-associated (MMD)* gene may reduce ion channel protein synthesis (99), whereas intron retention within adenosine deaminase (ADA) transcripts generates truncated proteins implicated in lysosomal dysfunction (100). These alterations collectively impair the ability of macrophages to eliminate intracellular MAP, observations derived from MAP-infected host-cell transcriptomic datasets (98)(**Figure 5**). Such macrophage functional defects support bacterial persistence and granulomatous inflammation in ruminant JD (27).

**Figure 5:**
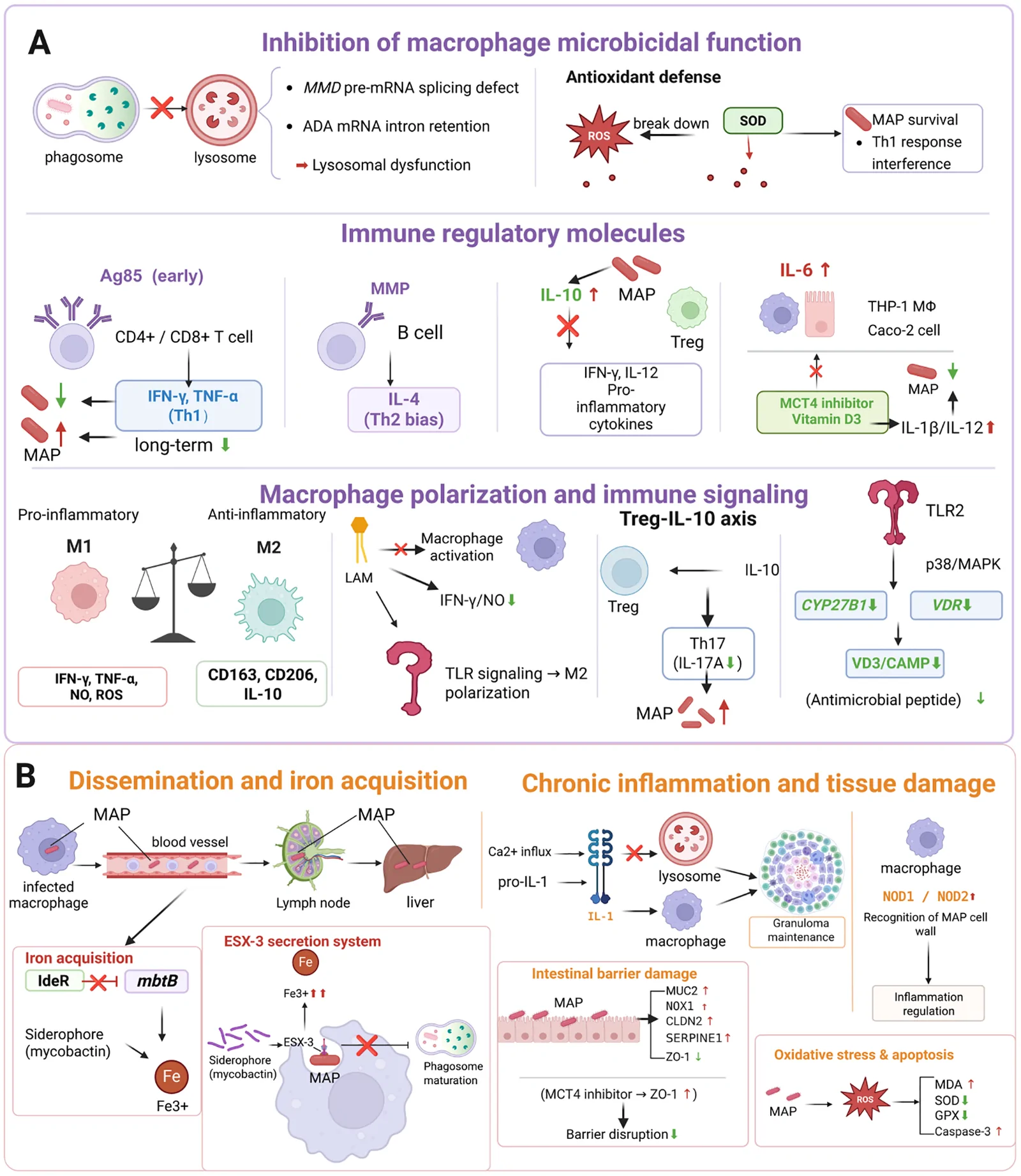
MAP-mediated immune evasion, dissemination, and pathological injury. **(A)** Immune evasion: MAP impairs the microbicidal function of macrophages through phagosome maturation defects and potent antioxidant defenses. Superoxide dismutase (SOD) scavenges reactive oxygen species (ROS), protecting MAP from oxidative damage and suppressing Th1-mediated immunity. Early Ag85 triggers protective Th1 responses, whereas mycobacterial major membrane protein (MMP) and interleukin-10 (IL-10) drive Th2/Treg polarization and immune tolerance. Lipoarabinomannan (LAM) promotes anti-inflammatory M2 macrophage polarization. The Toll-like receptor 2–p38/mitogen-activated protein kinase (TLR2–p38/MAPK) axis inhibits *CYP27B1/VDR* expression, reducing vitamin D-dependent antimicrobial peptide synthesis. **(B)** Systemic dissemination and chronic pathogenesis: Following intestinal entry, MAP replicates within macrophages, disseminates via the lymphatic system to mesenteric lymph nodes, and then hematogenously spreads to the liver, spleen, and other systemic organs. MAP acquires iron via the iron-dependent repressor (IdeR)–*mbtB*–mycobactin axis and ESX-3 secretion system to sustain intracellular survival. Chronic granuloma formation is initiated by sustained MAP-induced inflammation: impaired lysosomal function prolongs pro-IL-1 maturation and IL-1 release, recruiting monocytes, macrophages and lymphocytes to infection sites. Persistent antigen stimulation and NOD1/NOD2-mediated pattern recognition maintain granuloma structure, walling off MAP while driving chronic inflammation. Concurrently, intestinal barrier disruption (upregulated MUC2/NOX1/SERPINE1/CLDN2 and downregulated ZO-1), oxidative stress (elevated MDA and reduced SOD/GPX), and apoptosis contribute to irreversible tissue damage.

MAP infection also induces oxidative stress within host cells while simultaneously activating bacterial antioxidant defense systems. MAP-derived superoxide dismutase (SOD) neutralizes the reactive oxygen species (ROS) generated during the macrophage respiratory burst, thereby protecting the bacterium from oxidative damage (101). Moreover, SOD may interfere with protective T helper 1 (Th1) immune responses (83), further facilitating persistent infection, effects supported by MAP recombinant-protein and infection-based experimental data (102)(**Figure 5**).This antioxidant activity favors sustained intracellular MAP survival during JD.

#### 4.3.2 Manipulation of immune-regulatory molecules

During early infection, Ag85 can stimulate CD4^+^ and CD8^+^ T cells to produce Th1-associated cytokines such as interferon-γ (IFN-γ) and tumor necrosis factor-α (TNF-α), restricting bacterial proliferation (83). However, during chronic infection, MAP may suppress Ag85-mediated Th1 responses (102), ultimately leading to immune exhaustion and persistent infection establishment, these findings are derived from *in-vitro* assays using recombinant MAP-Ag85 antigen and bovine immune-cell culture infection experiments (27, 103), (**Figure 5**).This shift away from protective Th1 immunity facilitates sustained MAP persistence and granulomatous inflammation in ruminant JD.

The MMP promotes T helper 2 (Th2)-biased immune responses by stimulating B-cell proliferation and interleukin-4 (IL-4) secretion (104) while suppressing protective Th1 immunity (83). Elevated anti-MMP antibody levels correlate with clinical disease progression and may contribute to late-stage immunopathology (80), these observations come from *in-vitro* bovine cell stimulation assays and serological profiling of MAP-infected cattle (102, 104)(**Figure 5**). Such Th2-skewed immune responses exacerbate immunopathology during progressive JD in cattle.

MAP infection also induces increased secretion of interleukin-10 (IL-10), which is derived primarily from macrophages and regulatory T cells (Tregs). IL-10 suppresses proinflammatory cytokines such as IFN-γ and IL-12, creating an anti-inflammatory environment favorable for intracellular survival (105), these cytokine profiles were characterized using MAP-infected THP-1 macrophage and bovine primary macrophage *in-vitro* infection models (106). Concurrently, interleukin-6 (IL-6) is highly expressed in MAP-infected THP-1 macrophages and Caco-2 epithelial cells (107). Inhibition of monocarboxylate transporter 4 (MCT4) reduces IL-6 expression (108), whereas treatment with 1,25-dihydroxyvitamin D3 (1,25(OH)_2_D_3_) suppresses IL-6 while enhancing IL-1β and IL-12 production, thus strengthening antimicrobial responses (109)(**Figure 5**). This IL-10/IL-6 cytokine imbalance shapes the inflammatory microenvironment driving granulomatous enteritis in JD; similar cytokine dysregulation is also seen in CD intestinal lesions, yet it cannot be exclusively attributed to MAP infection in human patients.

#### 4.3.3 Immune cell polarization and functional interference

MAP preferentially drives macrophage polarization toward an anti-inflammatory M2 phenotype characterized by the expression of CD163, CD206, and IL-10 (110), rather than the proinflammatory M1 phenotype associated with IFN-γ and TNF-α production (111); these polarization signatures are observed from *in-vitro * infection assays using primary bovine macrophages and tissue immunohistology from MAP-infected cattle (112). Lipoarabinomannan (LAM) further suppresses macrophage activation by reducing IFN-γ and nitric oxide (NO) production (113) and promoting Toll-like receptor–mediated M2 polarization (111), weakening host antibacterial immunity; these functional outcomes are obtained from assays with purified MAP-derived LAM stimulating bovine macrophage cultures (113)(**Figure 5**). This M2-skewed macrophage phenotype blunts bactericidal responses and sustains intracellular MAP replication, supporting granuloma progression during ruminant JD (112); comparable M2-shifted macrophage profiles are seen in CD intestinal lesions, yet direct human-tissue evidence linking this phenotype to MAP infection remains absent.

Tregs and the IL-10 axis also contribute substantially to immune evasion. Dysregulated Treg-dependent IL-10 signaling may lead to aberrant T helper 17 (Th17) cell responses, including IL-17A production and mucosal pathogen clearance, indirectly facilitating MAP persistenc; these immune-cell observations are documented in MAP-infected bovine primary immune-cell culture systems (105) (**Figure 5**). Such Treg-Th17 axis disturbance exacerbates immune dysregulation in JD granulomatous enteritis.

Addition, MAP activates the Toll-like receptor 2 (TLR2)–p38/MAPK signaling pathway, resulting in downregulation of *CYP27B1* and *vitamin D receptor (VDR)* expression (109), reduced synthesis of active vitamin D3, and decreased production of the antimicrobial peptide CAMP (114); these molecular changes are characterized by MAP infection and pharmacological inhibitor assays in macrophage cell models (109). Pharmacological inhibition of the p38/MAPK pathway restores *CYP27B1*, *VDR*, and CAMP expression while attenuating inflammatory responses (109)(**Figure 5**). Suppression of vitamin-D-dependent antimicrobial defenses favors MAP intracellular survival within macrophages in JD. Though impaired vitamin-D-related antimicrobial pathways are also reported in CD patients (115), it cannot be ascribed specifically to MAP infection in human tissues.

### 4.4 Proliferation and dissemination

Following the establishment of intracellular persistence, MAP is disseminated by migrating macrophages to distant organs such as mesenteric lymph nodes and the liver, where it continues to proliferate under conditions of nutritional deprivation and immune pressure.

#### 4.4.1 Dependence on iron acquisition

Owing to evolutionary loss of portions of its siderophore system, MAP relies heavily on host-derived iron sources, including transferrin and lactoferrin. Expression of the mycobactin synthesis gene *mbtB* is controlled by the iron-dependent regulator (IdeR). These iron-responsive regulatory profiles are captured by *in-vitro* iron-limited transcriptomic and proteomic profiling of MAP, whereas functional assessment of the sheep-specific IdeR-R91G substitution is derived from heterologous reporter assays in *Mycobacterium smegmatis*, as corresponding IdeR knockout experiments have not been performed directly in MAP (116, 117)(**Figure 5**).Under iron-limited conditions, IdeR-mediated repression is relieved, promoting mycobactin synthesis and facilitating iron scavenging from host tissues. Of interest, a sheep-derived MAP IdeR mutant carrying the R91G substitution may alter iron storage regulation mediated by *bfrA*, potentially contributing to host adaptation and pathogenic divergence. This IdeR-driven iron-scavenging system, inferred from *in-vitro* omics data, is presumed to support intracellular replication and systemic organ dissemination during ruminant JD.

#### 4.4.2 Coordination between ESX-3 and iron metabolism

The type VII secretion system 3 (ESX-3), is a member of the mycobacterial type VII secretion system family. Transcriptomic data show that genes within the ESX-3 locus are significantly up-regulated under iron-limited *in-vitro* culture conditions in MAP, suggesting a potential link between ESX-3 and bacterial iron homeostasis (116). Most mechanistic interpretations regarding ESX-3-dependent iron acquisition are inferred from functional studies of *Mycobacterium tuberculosis* (MTB). In MTB, ESX-3 cooperates with the mycobactin biosynthesis machinery to assemble an efficient iron-acquisition and transmembrane-transport axis, and loss-of-function mutations in ESX-3 lead to growth defects that can be rescued by exogenous iron or zinc supplementation (118). Effectors secreted by ESX-3 are also hypothesized to interfere with host immune recognition and phagosome maturation to promote intracellular survival, a function that has been characterized primarily in MTB. To date, no targeted-deletion mutants of the ESX-3 gene cluster have been constructed and phenotypically assessed in MAP. Accordingly, genetic evidence confirming the essentiality of ESX-3, its functional redundancy with the *mbtB*/IdeR iron-regulatory circuit, and its direct *in-vitro* or *in-vivo* virulence-associated phenotypes in MAP remains absent. Therefore, current models for MAP ESX-3 function rely largely on extrapolation from homologous MTB research. If ESX-3 fulfils analogous iron-acquisition and immune-modulating roles in MAP, it could hypothetically support intracellular persistence and systemic dissemination during ruminant JD (**Figure 5**).

### 4.5 Chronic inflammation and pathological lesions

Persistent MAP infection ultimately results in chronic intestinal and systemic pathology, characterized primarily by granuloma formation, chronic diarrhea, and progressive wasting.

#### 4.5.1 Formation and maintenance of granulomas

Phagosomal acidification depends on extracellular calcium influx, which also participates in the conversion of pro–IL-1 to mature IL-1. As a key cytokine, IL-1 not only recruits macrophages to infection sites but is also essential for granuloma formation and maintenance. By suppressing lysosomal function through mechanisms such as abnormal pre-mRNA splicing and consequent *MMD*/ADA dysfunction, MAP prolongs inflammatory activity and promotes persistent granulomatous lesions; these splicing alterations are identified from transcriptomic profiling of MAP-infected macrophage cultures (119)(**Figure 5**). This mechanism sustains granuloma development in ruminant JD. While granulomatous injury is also the hallmark of CD intestinal pathology, it remains difficult to disentangle whether comparable splicing perturbations seen in human lesions stem from MAP infection or from general inflammatory bowel disease-intrinsic signalling.

LAM-mediated suppression of macrophage activation, reduction in IFN-γ and NO production, and induction of M2 polarization further impair bacterial clearance within granulomas, sustaining the chronic inflammatory microenvironment; these immunomodulatory effects are demonstrated using purified MAP-derived LAM in bovine macrophage *in-vitro* stimulation assays (28). Within JD granulomas, such macrophage reprogramming creates a permissive niche that favors ongoing MAP persistence.

In diffuse multibacillary lesions, nucleotide-binding oligomerization domain-containing protein 1 (NOD1) and nucleotide-binding oligomerization domain-containing protein 2 (NOD2) are highly expressed in macrophages and may regulate inflammatory responses through recognition of MAP cell wall components. In contrast, focal and diffuse paucibacillary lesions exhibit minimal NOD1/NOD2 expression, suggesting a close relationship between these pathways and lesion severity; these distinct expression profiles come from immunohistological profiling of intestinal biopsies from naturally infected cattle (120) (**Figure 5**). NOD-driven inflammatory signalling shapes lesion heterogeneity across different pathological forms of JD. Notably, NOD2 gene variants represent a well-established human CD susceptibility locus; however, existing human tissue datasets have not clarified whether MAP antigens engage NOD1/NOD2 signalling to amplify inflammatory responses in CD patients (**Figure 5**).

#### 4.5.2 Molecular determinants of tissue injury

Truncated mutants of the mycobactin synthesis gene *mbtA* exhibit defective siderophore production and become entirely dependent on host-derived iron sources. This metabolic restriction appears to reinforce chronic infection phenotypes characterized by impaired *in vitro* growth but enhanced persistence within the host; this reasoning stems from MAP genome-sequence observation, as targeted functional validation of truncated mbtA has not been completed in MAP knockout strains (121). Such iron-dependency is hypothesized to support long-term intracellular survival during JD.

MAP infection also induces oxidative stress-associated tissue injury, as evidenced by elevated levels of malondialdehyde (MDA) in the serum and intestinal mucosa, reduced activity of antioxidant enzymes such as SOD and glutathione peroxidase (GPX), and increased expression of caspase-3, indicating concurrent activation of apoptotic pathways. Of note, antioxidant enzyme activity remains relatively elevated in mesenteric lymph nodes, suggesting the occurrence of compensatory protective responses; these biomarker profiles are derived from serum and tissue measurements in naturally MAP-infected goats (122) (**Figure 5**). Oxidative damage and apoptosis constitute key drivers of mucosal tissue destruction in JD. Although oxidative stress and apoptosis are also well-documented pathological features in CD, multiple inflammatory triggers in human gut render it impossible to attribute these changes specifically to MAP exposure.

Moreover, MAP infection upregulates epithelial injury markers, including mucin 2 (MUC2), NADPH oxidase 1 (NOX-1), serpin family E member 1 (SERPINE1), and claudin-2 (CLDN2), while reducing the expression of the tight junction protein zonula occludens-1 (ZO-1/TJP1). The inhibition of MCT4 restores ZO-1 expression and alleviates epithelial barrier dysfunction; these barrier-perturbing signatures were characterized using MAP-challenged Caco-2 epithelial *in-vitro* cell infection assays (108) (**Figure 5**). Disrupted epithelial integrity is a prominent pathological outcome of JD. Intestinal tight-junction defects are also central to CD pathogenesis, but the MCT4-ZO-1 regulatory axis has not been validated in primary human intestinal biopsies (**Figure 5**).

Overall, MAP pathogenesis represents a highly coordinated, multistage process involving complex interactions between bacterial virulence determinants and host immune responses. During invasion and colonization, MAP exploits FAP and key virulence factors such as Mce1A/Mce1D and Ag85 to penetrate the intestinal mucosal barrier and invade epithelial cells. Following intracellular entry, MAP constructs a robust protective cell wall enriched with mycolic acids and GPLs while secreting effector proteins such as PknG and ESX-3 to block phagosome maturation and resist lysosomal killing. To withstand host immune pressure, MAP undergoes extensive metabolic reprogramming mediated by factors such as *relA* and *gcpE* while simultaneously manipulating host signaling pathways such as the TLR2–p38/MAPK axis, inducing anti-inflammatory cytokines and promoting Th2-biased immune responses to facilitate immune evasion. These processes ultimately culminate in chronic granulomatous inflammation, oxidative tissue injury, and intestinal epithelial barrier failure. A comprehensive understanding of these mechanisms provides an essential theoretical framework for the development of novel targeted vaccines, early diagnostic biomarkers, and innovative therapeutic strategies (119) (**Table 2**).

**Table 2:** Key pathogenic mechanisms and potential translational applications of MAP infection.

Infection stage	Key factors	Mechanisms	Translational implications	Reference
Adhesion & Colonization	FAP, Mce1A/Mce1D, Ag85, MMP, MAP3464	Epithelial adhesion and intestinal translocation	Early biomarkers; Mce/Ag85-based vaccines	(14, 80, 81, 83, 85, 93)
Barrier Penetration	Mce1A/Mce1D, MMP, Ag85	Barrier disruption and cellular invasion	Anti-invasion targets	(14, 80, 81)
Intracellular Survival	Mycolic acids, GPLs, PknG, ESX-3,*relA*	Inhibition of phagosome–lysosome fusion; Macrophage survival	PknG-targeted therapy; Iron metabolism interference	(86–88, 91–93, 95, 116, 118)
Immune Evasion	*relA*, *gcpE*, IL-10, LAM, TLR2–p38/MAPK, Th2 response	Metabolic reprogramming; Suppression of inflammation; Immune persistence	p38/MAPK and MCT4 inhibitors; MDA and caspase-3 biomarkers	(92, 95, 104–106, 109–113)
Chronic Damage	IL-10,LAM,*mbtA*,*relA*,NOD1/2	Granulomatous inflammation; oxidative tissue injury; Epithelial barrier failure	Anti-inflammatory and antioxidant strategies	(92, 105, 108, 113, 119–122)

FAP, Fibronectin Attachment Protein; Mce1A, Mycobacterial Cell Entry Protein 1A; Mce1D, Mycobacterial Cell Entry Protein 1D; Ag85, Antigen 85 complex; MMP, Mycobacterial Major Membrane Protein; MAP3464, MAP_3464 protein; GPLs, glycopeptidolipids; PknG, serine/threonine protein kinase G; ESX-3, ESX secretion system 3; *relA*, stringent response regulator A gene; *gcpE*, geranylgeranyl pyrophosphate synthase gene; IL-10, interleukin-10; TLR2, Toll-like receptor 2; MAPK, mitogen-activated protein kinase; Th2, type 2 T helper cell; LAM, Lipoarabinomannan; *mbtA*, mycobactin biosynthesis protein A gene; MCT4, Monocarboxylate Transporter 4; MDA, malondialdehyde; caspase-3, Cysteinyl Aspartate Protease 3, Toll-like receptor 2, type 2 T helper cell; NOD1/2, Nucleotide-binding Oligomerization Domain-containing protein 1/2.

## 5 Challenges and perspectives

MAP infection is widespread in dairy herds, yet current diagnostic approaches for early and subclinical infection remain limited by suboptimal sensitivity and specificity and the absence of rapid, field-deployable detection platforms (72, 123). Reliable early diagnosis is critical for timely intervention before disease dissemination, thereby reducing economic losses and public health risks. Accurate genotyping is also essential for source tracing and precision-based prevention and control strategies (12, 76). Conventional detection relies predominantly on fecal culture, histopathology, or IS900-based PCR assays (72, 74). Future platforms may incorporate MAP-specific proteins such as P900 and host-derived biomarkers, including NOD2 and IL-6. In addition, optimization of recombinase-aided amplification with lateral-flow dipstick (RAA-LFD) technology may enable rapid, highly sensitive, field-deployable detection (123). Surveillance of MAP contamination in dairy products and environmental samples is increasingly regarded as a valuable, noninvasive indicator for public health risk assessment (12, 76). Evidence supports a causal association between MAP infection and chronic inflammatory diseases such as JD (70, 73). Moreover, the MAP-derived P900 protein may induce autoimmune responses in genetically susceptible hosts through molecular mimicry (74). Enhanced monitoring of MAP throughout food supply chains and environmental reservoirs may therefore help interrupt potential zoonotic transmission pathways from animals to humans (12, 76).

Advances in genomics, proteomics, and bioinformatics are accelerating MAP research by enabling whole-genome and pangenomic analyses across diverse MAP strains (118, 120), which facilitates the identification of novel vaccine candidates and therapeutic targets. Genes such as *MAV_2043*, encoding copper–zinc superoxide dismutase (Cu/Zn-SOD) (73, 120), and *relA* are attractive targets for live-attenuated vaccine (LAV) development for veterinary use. Attenuated MAP strains generated through *relA* or *MAV_2043* deletion have demonstrated promising immunogenicity and safety profiles in pre-clinical animal studies for the prevention of Johne’s disease in ruminants (118, 120). Multiepitope subunit vaccines targeting p22 and MAP3531c are also under pre-clinical evaluation. These vaccine candidates are currently intended solely for livestock JD control, with no application for human patients (70, 73).

For human-relevant interventions, combination therapies integrating rifamycin-class antibiotics with targeted inhibitors are also being explored for MAP-associated autoimmune diseases. Together, these findings suggest that targeting MAP-specific pathogenic pathways may provide effective alternatives: veterinary vaccination strategies for JD control in livestock, and pharmacological interventions for related human disorders, particularly given the limited efficacy and increasing resistance risks linked to conventional antibiotic therapies (70, 73).

With continued advances in multiomics, diagnostics, genetically engineered vaccines, and One Health frameworks, the understanding of MAP biology and pathogenesis is expected to deepen substantially, driving progress in integrated disease control and ultimately supporting sustainable livestock production and global public health security (72, 76, 118).

## 6 Conclusion

MAP, once regarded primarily as an animal pathogen, is increasingly recognized as a potential contributor to multiple chronic inflammatory and autoimmune diseases. Its pathogenicity is driven by a coordinated multistage process involving intestinal invasion, intracellular persistence, immune evasion, and chronic granulomatous inflammation, which ultimately cause progressive tissue injury. Although accumulating epidemiological and mechanistic evidence supports associations between MAP and diseases such as CD, MS, and T1DM, its definitive etiological role remains controversial. Future work should focus on elucidating host–pathogen interactions, molecular mimicry, immune evasion mechanisms, and metabolic adaptations, while also facilitating the development of sensitive diagnostic tools, vaccines, and targeted therapies. A deeper understanding of MAP biology and pathogenesis will not only contribute to improved control of JD but also provide new insights into the prevention and treatment of MAP-associated human diseases.

## Data Availability

The original contributions presented in the study are included in the article/supplementary material. Further inquiries can be directed to the corresponding author.
